# Rapid-Onset Opioids for Management of Breakthrough Cancer Pain: Considerations for Daily Practice

**DOI:** 10.3389/fpain.2022.893530

**Published:** 2022-05-26

**Authors:** Paolo Bossi, Yolanda Escobar, Federico Pea

**Affiliations:** ^1^Department of Medical and Surgical Specialties, Radiological Sciences and Public Health - Medical Oncology, ASST-Spedali Civili, University of Brescia, Brescia, Italy; ^2^Medical Oncology, Gregorio Marañón General University Hospital, Madrid, Spain; ^3^Department of Medical and Surgical Sciences, Alma Mater Studiorum, University of Bologna, Bologna, Italy; ^4^Clinical Pharmacology Unit, IRCCS Azienda Ospedaliero Universitaria Sant'Orsola, Bologna, Italy

**Keywords:** breakthrough cancer pain, fentanyl, management, rapid-onset opioids, personalized approach

## Abstract

**Background and Objective:**

Rapid-onset opioids (ROOs) are effective treatments for breakthrough cancer pain (BTcP) given their rapid onset of action and relatively short duration of analgesia. The aim of this article is to describe specific considerations for the use of ROOs in daily practice, focusing on dose titration and treatment of specific populations.

**Type of Review:**

We conducted a narrative review on the use of ROOs for BTcP. We selected papers according to the following search terms: “breakthrough cancer pain” and “rapid onset opioids”.

**Results:**

ROOs may be considered as the most suitable drugs to treat BTcP and can be used “on-demand”. Several fentanyl formulations are available and have been associated with control of BTcP and with improvement in quality of life. Various titration schemes have been used to optimize ROO dosing; however, a dose-proportional scheme could be considered safe and effective in most patients. Specific formulations may be more suitable for specific patient subgroups; for example, patients with oral mucositis may prefer intranasal to oral formulations. Moreover, elderly patients or those without caregivers should be clearly educated on the use of these formulations. A key element in achieving successful treatment of BTcP is awareness of the barriers to pain management, including poor overall assessment, patient reluctance to take opioids or report pain, and physician reluctance to prescribe opioids.

**Conclusion:**

A personalized approach is fundamental when prescribing a medication for BTcP, and careful attention should be given to drug choice and route of administration, and to the need for alternative therapeutic options.

## Introduction

While seemingly increased attention is being paid to adequate treatment of cancer pain, the proportion of cancer patients experiencing pain has changed little in recent years, with a high proportion still suffering from moderate to severe pain (1, 2). In addition to background cancer pain, in the last two decades much effort has been made to identify and treat breakthrough cancer pain (BTcP). BTcP can be broadly defined as pain that breaks through otherwise well controlled background cancer pain (3). Overall, the etiology of BTcP is likely similar to that of chronic cancer pain, and may be related to tissue destruction by the cancer, tumor treatment, and other cancer-related conditions (3). Episodes of BTcP are characterized by their rapid onset, high intensity, and short duration (4).

BTcP can be categorized into spontaneous and incident, where the former is idiopathic and the latter has a trigger such as movement or voiding (3). Incident BTcP can be further subclassified as: (i) volitional (e.g., brought on by walking); (ii) non-volitional (e.g., invoked by an involuntary act such as coughing); or (iii) procedural (e.g., resulting from a therapeutic intervention such as dressing a wound). BTcP is not a single well-defined entity, but rather a number of entities that present differently in each patient. Unfortunately, at present, BTcP remains an undertreated yet challenging entity to manage despite the availability of rapid and effective analgesics (5, 6).

Rapid-onset opioids (ROOs) have become a mainstay in the treatment of BTcP. In this article, we review the management of BTcP with the aim of providing specific considerations for daily practice, with a focus on dose titration and the treatment of specific patient groups. We performed a narrative review utilizing PubMed and Google Scholar Databases and identifying papers between 2000 and 2021, with the following search terms: “breakthrough cancer pain” and “rapid onset opioids”.

## Prevalence and Characteristics of BTCP

The prevalence of BTcP depends on the individual patient and the specific type of cancer, but it is broadly reported by 40–80% of patients. In a large, pooled analysis of 19 studies, BTcP was reported by 59.2% of patients (7). In an observational study involving 1,000 oncology patients, BTcP was reported by the vast majority, with 44% reporting incident pain, 42% spontaneous pain, and 14.5% a combination of both (4). BTcP has been reported in more than 89% of hospice patients with pain, and the prevalence rate among this patient population is generally higher than observed in outpatients (7, 8). These percentages are also reflected in a recent Spanish analysis, where, among 371 cancer patients, 38% reported episodic pain without a defined trigger and 49% described pain associated with a triggering event (9).

As far as the characteristics of BTcP are concerned, the Italian Oncologic Pain multiSetting Multicentric Survey (IOPS-MS) study showed that among 1,500 patients BTcP occurred with a mean of 2.5 episodes per day, had a mean intensity of 7.5 on a scale from 0 to 10, and a mean duration of 43 min. Moreover, BTcP was predictable in one-third of cases (10). Some subgroups of patients may be at higher risk of developing BTcP, as demonstrated in the sub-analysis of the IOPS-MS study (11). Patients with head and neck cancer had the highest number of episodes per day (2.8/day) (11). The episodes were linked to food/liquid ingestion, and thus were complicated by nutritional issues. Consequently, it is advisable to assess and monitor these patients in different ways. Regardless, BTcP may markedly interfere with daily activities in almost all patients, significantly impairing their quality of life (12).

## Roos for Treatment of BTCP

BTcP is characterized by rapid onset and short duration over a background of well controlled persistent pain. Oral morphine should not be considered for the treatment of unpredictable BTcP, as the time to pain relief is usually more than 30 min (13), so would not be effective in controlling idiopathic or non-volitional incident pain (14). Conversely, ROOs may be considered as the most suitable drugs to counteract BTcP (15, 16). ROOs have a typical onset of action within minutes and a duration of action of only few hours (14). ROOs can be consistently used “on demand”, which is valuable considering that BTcP is often unpredictable (14, 15). On the other hand, either ROOs or oral morphine could have a role when the BTcP could be foreseen (this indication is not included in the summaries of product characteristics), such as in the case of procedural or volitional BTcP, although data and studies on the efficacy, safety and cost-effectiveness of these treatments when used in this manner are lacking and further research is required (17).

A number of formulations of fentanyl are currently approved for the treatment of BTcP. These include oral transmucosal fentanyl citrate (lollipop format), fentanyl buccal tablet, fentanyl buccal soluble film, and sublingual fentanyl, in addition to nasal transmucosal formulations (18). A large number of clinical trials and meta-analyses have documented the efficacy of fentanyl formulations in controlling BTcP, with a rapid onset of pain relief (within 15 min) and superiority over oral morphine (14, 19). In a meta-analysis by Zeppetella et al., the difference in pain intensity control compared with placebo 15 min after intake was greater with all formulations of fentany than with other BTcP medications (20). Moreover, a reduction in the number of BTcP episodes was documented and patient satisfaction was rated as excellent or good (21, 22). Patient satisfaction was also rated as high with fentanyl pectin nasal spray (23). While some reports suggested that fentanyl buccal tablet may have some advantages over other fentanyl formulations, this has not been consistently demonstrated (24). Compared with subcutaneous morphine, fentanyl sublingual tablets did not show non-inferiority in a randomized double-blind trial; however, most patients preferred the sublingual route of administration (25). Importantly, rapid-onset fentanyl has also been associated with significant improvement in health-related quality of life. In the BEST observational study, among 154 patients in palliative care units with stable control of background pain who received transmucosal fentanyl for BTcP, almost all physical and emotional domains measured with the European Organization for Research and Treatment of Cancer (EORTC) Quality of Life Questionnaire Core 15 Palliative Care (QLQ-C15-PAL) showed significant improvement; exceptions were nausea, vomiting, and dyspnea (26).

## Dose Titration

It is widely believed that the dose of all opioid rescue medications should be determined by individual titration (27). When testing the starting dose, four different scenarios may be considered. If the pain is well controlled without adverse effects, then the same dose may be used for future episodes. If the pain is well controlled but adverse effects occur, the dose should be decreased. If the pain is uncontrolled and no adverse effect occurs, the dose should be increased. Lastly, if the pain is uncontrolled and adverse effects appear, drug treatment should be changed.

The use of a dose titration scheme and the titration protocol itself have varied somewhat among different studies. In some trials, the effective dose was considered as that allowing successful treatment of two consecutive episodes of BTcP (28).

Furthermore, there has been some discussion about the use of proportional dosing (29, 30). It has been suggested that proportional dosing may have several benefits, such as being applicable across all doses of baseline opioid treatment and in a variety of settings (29). Proportional dosing has also been supported by a number of studies, including some conducted in home settings and in highly tolerant patients, and is generally well tolerated (31, 32). Indeed, doses that are proportional to the baseline opioid regimen used for background pain appear to be both effective and safe in most patients (33). This has been confirmed in a relatively recent study in which administration of fentanyl buccal soluble film at proportional doses based on current regimen for baseline pain resulted in only 12% of patients requiring dose titration in the per-protocol population (34).

## Treatment Considerations in Specific Subpopulations

Each of the available formulations of rapid-onset fentanyl has characteristics and advantages that may render it more suited for some specific patient groups as highlighted below (35). However, it should be acknowledged that we cannot fully dissect all the factors possibly leading to different responses to ROOs. As an example, the sex and gender differences in ROOs use have not been fully investigated, while some works underlined how these differences are important in pain perception (36).

### Mucositis or Oral Intolerance

Many patients undergoing radiotherapy and/or chemotherapy will experience painful oral mucositis to different degrees (37). The rate of fentanyl absorption from the buccal tablet was similar in patients with or without mucositis (38). Thus, this may definitely be a valid option in patient with mucositis who are still able to take oral formulations. For patients who find oral formulations of fentanyl difficult to use (35), an intranasal formulation may represent a more suitable and better accepted option (39). During curative radiotherapy, patients with head and neck cancer (irrespective of whether or not they are receiving chemotherapy) have been shown to benefit from the use of intranasal fentanyl, with a decrease in the frequency of incidental BTcP caused by food/liquid swallowing (40). Very recently, high rates of patient satisfaction were also shown with an intranasal formulation of fentanyl in patients undergoing radiotherapy who are at high risk of mucositis (23).

### Patients Without Caregivers

In patients without caregivers, the choice of formulation obviously depends on the patient's preferences and functional abilities (35). All oral transmucosal fentanyl formulations can be used by those who find it difficult to swallow. However, it has been noted that the drug and delivery system could be ingested and should thus be avoided in some cases (35). In addition, there is the potential risk of dental decay with the “lollipop” formulation. Intranasal formulations can be proposed, depending on the patient's dexterity.

### Elderly

The proportion of elderly people who experience pain is already high in those without cancer, but increases further in the presence of cancer (41, 42). Treatment of BTcP in the elderly is challenging due to comorbidities, cognitive decline, polypharmacy, and decreased hepatic and renal function. It is important to carefully titrate the background pain medication, and ROOs may be considered for BTcP. Some authors have advocated that other medications such as non-steroidal anti-inflammatory drugs may be an option for BTcP in the elderly (42). It has been suggested that the initial dose of BTcP medications should be one-sixth of the patient's total daily opioid, and can then be titrated (42). It is clear that BTcP treatment decisions in elderly patients should be highly personalized, taking into account the global profile of each individual patient. In 2019, using the Delphi process, a Spanish group issued a set of recommendations on the management of BTcP in the elderly (43). There was a high degree of consensus that transmucosal fentanyl coupled with a “start slow and go slow” dosing method is the most suitable treatment approach for BTcP in this patient group.

### Patients on Polypharmacy

Many patients with cancer are likely to be treated for comorbidities and/or may receive anti-cancer medications and, as such, polypharmacy is common in this group. Fentanyl is metabolized mainly *via* the human cytochrome P450 3A4 isoenzyme system (CYP3A4), and thus potential drug-drug interactions may occur in patients cotreated with drugs that are CYP3A4 inhibitors or inducers (44). Use of fentanyl with strong or moderate CYP3A4 inhibitors may result in increased fentanyl plasma concentrations. As such, patients receiving such co-medications should be carefully monitored for adverse events.

In patients receiving CNS depressants, the dose and duration of concomitant fentanyl use should be limited. Moreover, the concomitant use of partial opioid agonists/antagonists (e.g., buprenorphine, nalbuphine, and pentazocine) should be avoided. Lastly, it has been reported that drug interactions are unlikely to alter the onset or duration of analgesia, but they may affect its duration (44).

Psychological symptoms such as anxiety and depression are common in cancer patients and have a significant negative impact on daily activities and functioning (45). While adequate treatment of BTcP can improve the patient's health-related quality of life (26), it is nonetheless clear that a substantial proportion of patients will still suffer from a range of psychological symptoms that warrant treatment. When administering psychotropic medications to patients with cancer, careful attention should be paid to the side effect profile and potential for drug interactions (45). Caution is needed during co-administration of fentanyl-based ROOs with a serotoninergic agent because of the potential for interactions. Prescribers should be aware that selective serotonin re-uptake inhibitors, serotonin norepinephrine re-uptake inhibitors, and monoamine oxidase inhibitors (MAOIs) may increase the risk of serotoninergic syndrome. Moreover, fentanyl-based formulations should be avoided in patients who have received MAOIs in the previous 14 days, since this may lead to severe and unpredictable potentiation of the MAOI effect. Before prescribing a ROO or a psychotropic agent, the patient's current medication regimen should be carefully reviewed to ensure that no risk of drug interaction exists.

### Idiopathic vs. Incident Pain

While idiopathic BTcP is unpredictable, incident BTcP can be predicted. Any episode of pain that is linked to diagnostic or therapeutic procedures may be considered as BTcP. Often this pain is underestimated because diagnostic procedures and therapies are considered the main objective, so providers may give less consideration to procedure-related symptoms experienced by patients. Examples of these types of pain have been reported in detail, and can occur during procedures such as lumbar puncture, bone biopsy, endoscopy, and tumor embolization (46). Since the BTcP associated with these procedures can be predicted, providers should be aware of them and try to minimize patient discomfort. Thus, by knowing that some movements or procedures may trigger BTcP in some patients, administration of rescue medication before starting them could prevent triggering of BTcP.

## The Role of Physician's Education

As in all fields of medicine, adequate knowledge is key in achieving successful treatment. Pain is a complex issue, and its treatment warrants a multidisciplinary approach (47). Physicians need to be aware of potential barriers to effective pain management, such as poor overall assessment, patient reluctance in taking opioids or in reporting pain, and physician reluctance in prescribing opioids (48).

A survey of eight clinical vignettes among 570 oncologists highlighted knowledge deficiencies in the management of cancer pain, underscoring the need for improving knowledge on pain management through educational activities (49). Likewise, in a survey of 2000 oncologists in the USA in 2011, only 10% of oncologists said that they would have recommended a ROO for BTcP (50). In another survey of Korean physicians in 2014, it was noted that knowledge of guidelines for the control of cancer pain was associated with improved pain management, although general compliance with guidelines was lacking (51). The lack of compliance with guidelines on BTcP was also highlighted in a more recent survey of oncologists in Spain, in which 99% agreed that guidelines provide the best scientific evidence, but a lower percentage of prescribers (76–92%) were compliant with those guidelines (52). This suggests that educational activities may be helpful in improving the implementation of current guidelines. Other recent surveys of pain specialists in Spain showed that ROOs are still underused (53, 54).

While there is some evidence to suggest that overall knowledge is improving, gaps still remain and more education is needed in order to increase adherence to current guidelines and to optimize management of BTcP, especially considering that a number of valuable therapeutic options are currently available.

## Considerations for Daily Practice

A personalized approach is fundamental when prescribing a medication for BTcP, and careful attention should be given to drug choice and route of administration, and to the need for alternative therapeutic options. Clinicians should be confident that background pain medication has been optimized before prescribing any drug for BTcP. Attention should be given to the specific characteristics of the BTcP (onset, predictability, severity, and duration), possible clustering, underlying disease, adherence to medication regimens, and formulation preferences. In this regard, some patients may find inhalatory medications difficult or uncomfortable to use, and patients with severe mucositis may prefer to avoid oral formulations.

Any decision regarding the use of a specific rescue medication for treatment of BTcP, usually an ROO formulation, should be based on four factors: (1) the characteristics of the BTcP, including duration and time to peak intensity; (2) the drug's characteristics, attempting to match the pharmacokinetic profile to the patient's BTcP; (3) previous responses to opioid therapy (e.g., efficacy and tolerability); and (4) the patient's preference for route of administration. Additionally, more attention should be focused on the inappropriate use and misprescribing of all opioids. Patients must be evaluated for possible misuse, and be carefully assessed for risk factors of abuse and aberrant behavior (55). Some centers have adopted specific measures aimed at minimizing abuse (55).

Looking forward to the future, recent studies have suggested that the optimal dose of BTcP opioids might depend on the dose of background opioids (56). If confirmed, this would allow prescribers to personalize therapy for BTcP based on the characteristics of each patient and further define a more precise strategy for its management.

There are still a few unanswered questions regarding the management of BTcP and the possible impact of an optimal pain control on anticancer treatment tolerability and treatment intensity. It has not still been clearly defined whether a better pain management, including background and breakthrough cancer pain, could translate into greater adherence to oncologic therapy and definitely into higher possibility of obtaining tumor response.

Another debate regards the possible immune depressing activity of opioids, thus potentially harming cancer patients under immune checkpoint inhibitors (ICI) treatment. This possible interaction has been demonstrated both as a repressive effect on the immune system and as a dysregulation of gut microbiota leading to an indirect effect on ICI effectiveness (57). While these aspects need to be further elucidated, no study has specifically investigated the impact of ROOs on the immune system of patients ongoing anticancer treatments.

## Conclusions

ROOs have proven efficacy in the treatment of BTcP and they are available in a variety of formulations including for administration *via* the oral (buccal and sublingual) and nasal transmucosal routes, thus allowing for a personalized approach when prescribing these medications. Careful attention should be paid to drug choice, route of administration and dose titration, as well as the need for alternative therapeutic options, as the tailoring of treatment approaches is essential to allow patients with BTcP to experience good pain control and thus improved quality of life. There are still a few unanswered questions about the interaction between opioid use and anticancer treatments that need to be fully investigated.

## Author Contributions

The paper was designed by and written with the contribution of all the authors. All authors discussed the content, commented on all drafts of the manuscript, contributed to the article, and approved the submitted version.

## Funding

This study was supported by an unrestricted grant from Angelini Pharma. The funder was not involved in the study design, collection, analysis, interpretation of data, the writing of this article or the decision to submit it for publication.

## Author Disclaimer

The authors are fully responsible for all content and editorial decisions for this manuscript.

## Conflict of Interest

PB has participated in advisory boards or received conference honoraria from Angelini and Molteni. YE has participated in advisory boards or received conference honoraria from Angelini, Kiowa Kirin, Grunenthal, Ferrer, Mundipharma, Vifor Pharma, Tesaro, Mylan, Merck Serono, Merck Sharp & Dome, BMS, Sanofi Aventis, and Astra Zeneca. FP has participated in the speaker bureau for Angelini, Basilea Pharmaceutica, Gilead, Hikma, Merck Sharp & Dohme, Nordic Pharma, Pfizer, and Sanofi Aventis, and in advisory boards for Angelini, Basilea Pharmaceutica, Correvio, Gilead, Merck Sharp & Dohme, Nordic Pharma, Novartis, Pfizer, and Thermo-Fisher.

## Publisher's Note

All claims expressed in this article are solely those of the authors and do not necessarily represent those of their affiliated organizations, or those of the publisher, the editors and the reviewers. Any product that may be evaluated in this article, or claim that may be made by its manufacturer, is not guaranteed or endorsed by the publisher.
